# Associations between Hip Fracture Operation Waiting Time and Complications in Asian Geriatric Patients: A Taiwan Medical Center Study

**DOI:** 10.3390/ijerph18062848

**Published:** 2021-03-11

**Authors:** Ching-Yi Shen, Chien-Han Hsiao, Weide Tsai, Wen-Han Chang, Tse-Hao Chen

**Affiliations:** 1 Department of Emergency Medicine, Mackay Memorial Hospital, Taipei 104, Taiwan; ellen8306003@gmail.com (C.-Y.S.); weidetsai@gmail.com (W.T.); branden888@gmail.com (W.-H.C.); 2 Department of Medicine, Mackay Medical College, New Taipei City 252, Taiwan; 3 Department of Linguistics, Indiana University, Bloomington, IN 47405, USA; chiehsia@indiana.edu; 4 MacKay Junior College of Medicine, Nursing, and Management, Taipei 112, Taiwan; 5 Institute of Mechatronic Engineering, National Taipei University of Technology, Taipei 106, Taiwan

**Keywords:** geriatric, hip fracture, operation waiting time, Asian, complication

## Abstract

Early surgical intervention in hip fractures is associated with lower complications. This study aimed to determine the appropriate operation time among Asian geriatric patients. The data of 1118 elderly patients with hip fracture at Mackay Memorial Hospital from 1 January 2011, to 31 July 2019, were retrospectively examined. Association between operation waiting time and the occurrence of complications was calculated using a cubic spline model. Significantly increased incidence of pneumonia, myocardial infarction, and heart failure was observed in 30 and 90 days when the patient’s surgical waiting time exceeded 36 h. The incidence rates of pneumonia across the early and delayed groups within 30 and 90 days were 4.4% vs. 7.9%, and 6.2% vs. 10.7%, those of myocardial infarction were 3.0% vs. 7.2%, and 5.7% vs. 9.3%, and those of heart failure were 15.2% vs. 26.8%, and 16.2% vs. 28.5%. Deep vein thrombosis and pulmonary embolism were not associated with surgical delay. The overall 30-day mortality rate was 5.4%, and no significant difference was observed when the surgical waiting time exceeded 36 h. In summary, operation waiting time exceeding 36-h was associated with increased rates of pneumonia, myocardial infarction, and heart failure in Asian geriatric patients undergoing hip fracture surgery.

## 1. Introduction

Hip fracture is a devastating injury with complications that can lead to serious morbidity and mortality. A previous study projected an increased rate of hip fracture in Asian countries of approximately 2.56 million victims in 2050 [1]. Among the countries, Taiwan has been estimated to have the highest incidence rate of hip fracture. Along with the increase of hip fracture incidence in Asian countries, studies have also predicted an increase in medical cost and healthcare system burden among Asian countries [1,2,3]. Therefore, this condition should be considered a top medical priority. Some studies have investigated the association between operation waiting time and complication risks, and found that early surgical intervention of hip fracture is associated with a lower complication rate [4,5,6,7]. Despite efforts to lower the risk of medical complications accompanying hip fracture, there is no consensus on the reasonable operation waiting time in the current literature. Various operation waiting time recommendations have been advised. The European Society of Trauma and Emergency Surgery suggested an operation waiting time of 24 h [8]. In contrast, the American Academy of Orthopedic Surgeons recommended that the surgery be performed within 48 h [9]. Notwithstanding this valuable information, no practical guideline on operation waiting time for the Asian population has been proposed. Moreover, among the sufferers of hip fracture, geriatric patients are more vulnerable to severe complications. Therefore, we aimed to determine the appropriate operation time in Asian geriatric patients in this study.

## 2. Materials and Methods

The design and execution of this retrospective study was approved by the Institutional Review Board of MacKay Memorial Hospital (19MMHIS334e).

### 2.1. Patients

**Figure 1:**
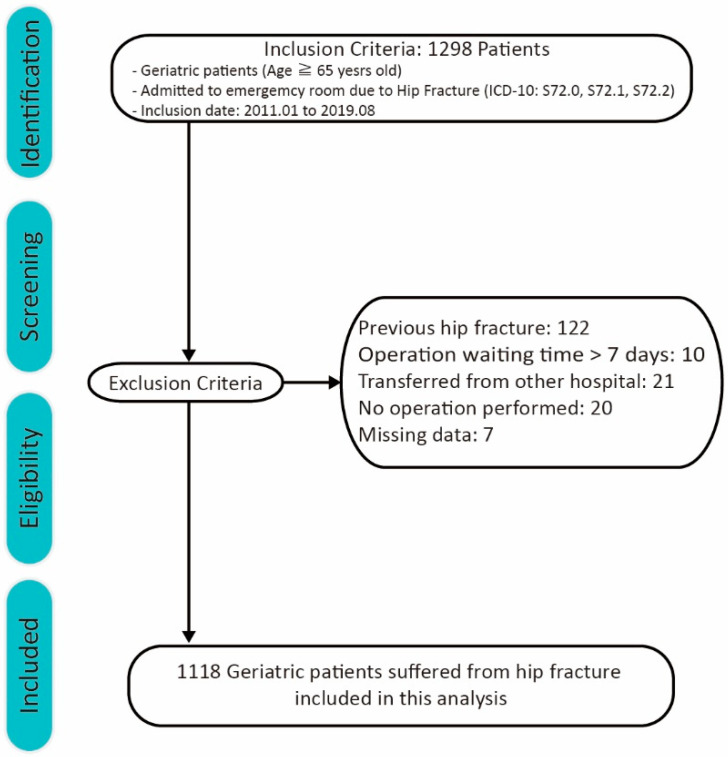
Flow chart of patient inclusion.

This study collected data on geriatric patients who were at least 65 years of age and visited our emergency room (ER) between January 2011 and August 2019. The data of the hip fracture geriatric patients were retrieved from our electronic medical record database using the International Classification of Diseases 10th Edition Clinical Modification (S72.0, S72.1, and S72.2). A total of 1298 geriatric patients were identified with the above criteria. The exclusion criteria were as follows: patients (i) with a record of previous hip fracture, (ii) who underwent an operation after 7 days, (iii) who were not diagnosed in our hospital, (iv) who did not receive intervention, and (v) whose data were missing (Figure 1). A total of 180 patients were excluded with the above criteria, and 1118 geriatric patients were included in the analysis.

### 2.2. Main Variable

The main independent variable is each patient’s operation waiting time. Operation waiting time is defined as the time between a patient’s arrival in the ER and the start of their surgery (in hours).

### 2.3. Covariates

Multiple covariates were taken into consideration in the statistical analysis, including baseline characteristics (i.e., age, gender, and hypertension and diabetes mellitus prevalence), biochemical parameters (i.e., admission hemoglobin, liver function, renal function (modification of diet in renal disease {MDRD} equation), and coagulation profile), ER Glasgow coma scale (GCS), triage level, and injury severity score (ISS). Additionally, factors such as ER visit day (i.e., weekdays and weekends), hip fracture type (i.e., femoral neck fracture, intertrochanteric fracture, and subtrochanteric fracture), operation type (i.e., arthroplasty and internal fixation), and operation time (i.e., working hours (08:00 to 16:59), evening hours (17:00 to 23:59), and overnight (00:00 to 07:59)) were coded (Table 1).

### 2.4. Statistical Analysis

The statistical software R (R-4.0.2) (R Core Team, Vienna, Austria) [10], development interface R studio (RStudio Team, Boston, MA, USA) [11], and data visualization package ggplot2 [12] were used for data analysis and visualization. The probability of complications as a function of operation delay was modeled with cubic splines with four knots [6,13], and the inflection points (in hours) of each type of medical complication were visualized. The software SPSS (version 26.0; SPSS Inc., Armonk, NY, USA) was used to calculate baseline cohort characteristics and outcomes [14]. We tested these characteristics using chi-square and independent *t* tests. Post-hoc analysis adjusting for gender, age, ISS, triage level, and GCS was conducted with a binary logistic regression. The threshold of statistical significance was set to a *p*-value of < 0.05.

### 2.5. Clinical Outcomes

**Figure 2:**
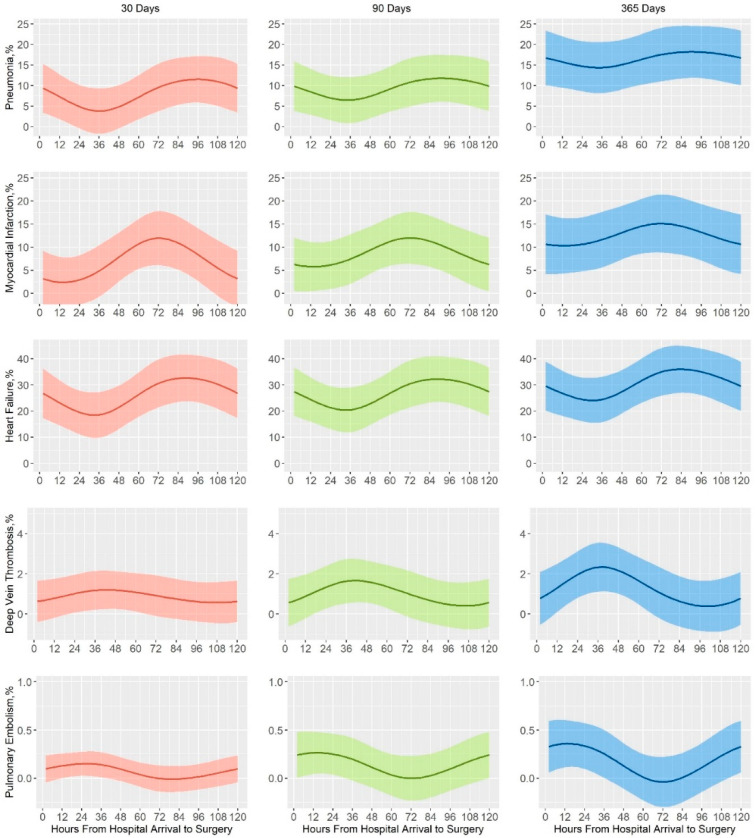
Probabilities (95% confidence intervals) modeled with cubic splines model. Probabilities of operation waiting time and each medical complication (pneumonia, acute myocardial infarction, heart failure, deep vein thrombosis, and pulmonary embolism) are presented.

The primary clinical outcomes included several major medical complications, such as pneumonia, acute myocardial infarction, heart failure, deep vein thrombosis, and pulmonary embolism within 30, 90, and 365 days (Figure 2). We identified early versus delayed groups with operation waiting time thresholds of 6, 12, 24, 36, and 48 h. Maximum area under the curve was calculated for each aforementioned threshold across medical complications to determine the boundary between the early and delayed surgical groups. A 30-day mortality rate was considered a secondary outcome. Mortality within 30 days after injury was a standard follow-up period for trauma based on the Utstein template [15]. A recent study also indicated that prolonging the follow-up period for more than 30 days increases the proportion of non-traumatic deaths [16].

## 3. Results

A total of 1118 geriatric patients who suffered from hip fracture were included in this study (Figure 1). The mean age of the patients was 80.81 ± 7.56 years, and 69% were women. The mean ER arrival to operation waiting time was 29.22 ± 19.55 h. The maximum area under the curve was 36 h across medical complications (Appendix A). Hence, we divided patients into the early and delayed surgical groups, with a threshold of a 36-h operation waiting time. With these criteria, 827 patients (73.9%) underwent early surgery, whereas 291 patients (26.1%) received delayed surgery.

The baseline characteristics of the early and delayed surgery groups showed no statistical difference in patients’ age (80.69 vs. 81.16 years, *p* = 0.519), gender (female percentage: 71% vs. 66%, *p* = 0.143), hypertension prevalence (36% vs. 37%, *p* = 0.67), hemoglobin level (11.76 vs. 11.33 mg/dL, *p* = 0.63), liver function (aspartate aminotransferase level: 28.56 vs. 30.13 IU/L, *p* = 0.28), renal function (glomerular filtration rate calculated using MDRD: 61.51 vs. 53.43 mg/dL, *p* = 0.94), and coagulation (international normalized ratio: 1.11 vs. 1.12, *p* = 0.76). However, the prevalence of diabetes mellitus was borderline higher in the delayed surgical group (38% vs. 45%, *p* = 0.042; Table 1).

ER characteristics showed no statistical difference in triage level (*p* = 0.69) and ISS (9.11 vs. 9.21, *p* = 0.43). In contrast, GCS unveiled a statically significant difference (14.96 vs. 14.86, *p* < 0.05) even though the ratings of both groups were close to 15 points. The other major difference was the patients’ ER visit days. Comparing the two groups, over 50% of the delayed group patients visited the ER on weekends (21.4% vs. 52.6%, *p* < 0.05). No significant difference was found in the patients’ fracture type (*p* = 0.428), operation type (*p* = 0.157), and operation time (*p* = 0.521; Table 1).

Outcome comparison between the early and delayed surgical groups is shown in Table 2. Increased pneumonia rate risks in 30 (4.4% vs. 7.9%, *p* < 0.05) and 90 days (6.2% vs. 10.7%, *p* < 0.05) were found in the delayed surgical group, with statistical significance. However, no statistical difference was found on the pneumonia rate in 365 days (12.8% vs. 17.2%, *p* = 0.065). Multivariate analysis adjusting for gender, age, ISS, triage level, and GCS revealed the same results. The incidence rates of acute myocardial infarction in 30 (3.0% vs. 7.2%, *p* < 0.05) and 90 days (5.7% vs. 9.3%, *p* < 0.05) were elevated, but this trend was not found in the incidence rate at 365 days (8.7% vs. 12%, *p* = 0.098). Multivariate analysis also confirmed this result. Escalated risk of heart failure was also found in 30 (15.2% vs. 26.8%, *p* < 0.05), 90 (16.2% vs. 28.5%, *p* < 0.05), and 365 days (19.6% vs. 32.6%, *p* < 0.05), with statistical significance. There were no statistical difference in the incidence rates of deep vein thrombosis in 30 (1.0% vs. 2.1%, *p* = 0.215), 90 (1.1% vs. 2.4%, *p* = 0.146), or 365 days (1.7% vs. 3.4%, *p* = 0.078) and of pulmonary embolism in 30 (0.4% vs. 0%, *p* = 0.572), 90 (0.5% vs. 0%, *p* = 0.578), or 365 days (0.6% vs. 0%, *p* = 0.184).

Chi-square analysis revealed no difference in the 30-day mortality rate between the early and delayed surgical groups (0.4% vs. 1.0%, *p* = 0.186). No difference was noticed in patients’ operation duration (115.4 vs. 114.9 min, *p* = 0.402) and operation blood loss (211.3 vs. 177.8 mL, *p* = 0.256). However, the statistical test showed a significantly prolonged length of hospitalization for the delayed group (7.4 vs. 9.6 days, *p* < 0.05; Table 2). Chi-square test of independence was calculated comparing the rates of mortality and morbidities in patients who underwent internal fixation vs. arthroplasty. The results suggested no significant difference in the 30-day mortality rates (*p* = 0.500), 30-day pneumonia rates (*p* = 0.592), 90-day pneumonia rates (*p* = 0.648), 30-day myocardial infarction rates (*p* = 0.236), 90-day myocardial infarction rates (*p* = 0.334), 30-day heart failure rates (*p* = 0.380), 90-day heart failure rates (*p* = 0.394), and 365-day heart failure rates (*p* = 0.193) across the two surgery types.

## 4. Discussion

To our knowledge, the current study is the first to analyze the medical complications in Asian geriatric patients who had hip fracture. By treating operation waiting time as a continuous variable and calculating the threshold of increased medical complication rate after hip fracture, instead of arbitrarily classifying patients into the early or delayed surgery groups, we were able to identify a threshold of 36 h; a point after which increases in several medical complications were observed.

Our study shows that after 36 h, there is a statistically significant increase in the risk of pneumonia, acute myocardial infarction, and heart failure in 30-day and 90-day follow-ups, and noted even in multivariate analyses. In the study by Pincus et al. [6], increased risks of pneumonia, acute myocardial infarction, deep vein thrombosis, and pulmonary embolism were noted after an operation waiting time threshold of 24 h [6]. The characteristics of our and Pincus et al.’s patients were similar [6]. Our patients had a mean age of 80.76 (vs. 80.81) years, a male-to-female ratio of 70% (vs. 69%), a femoral neck fracture percentage of 50% (vs. 51.7%), and an arthroplasty operation percentage of 38% (vs. 50%). Therefore, we would like to suggest that the difference in the thresholds of operation waiting time arose from the patients’ ethnicity, and recommend an operation waiting time threshold guideline of 36 h to be considered for the Asian population.

The other major difference was the incidence rates of deep vein thrombosis and pulmonary embolism. A study in Ontario between 2009 and 2014 revealed an elevated incidence rate of deep vein thrombosis and pulmonary embolism when operation waiting time exceeded 24 h [6]. However, a study in Japan between 2010 and 2014 did not find an increase in the risk of pulmonary embolism in elderly hip fracture patients who underwent surgery before, versus after, 2 days of waiting time [7]. In accordance with this study, our study also suggested that there is no increased risk of deep vein thrombosis or pulmonary embolism in patients with an operation waiting time exceeding 36 h. The overall incidence rate of deep vein thrombosis and pulmonary embolism was 1.5% (17/1118 patients) in a 30-day follow-up, which was comparable with the incidence rate of 1.7% in the SMART study [17]. This difference between the Western and Eastern populations could be attributed to the absence of some genetic factors in the Asian population. A former report indicated that the lack of thrombophilic polymorphisms combined with low prothrombotic risk factors led to low prevalence of deep vein thrombosis and pulmonary embolism in Asian patients undergoing total hip arthroplasty [18].

Prior studies were inconsistent on the mortality rates in the delayed surgical intervention group [6,7,19,20,21,22,23,24,25]. This inconsistency could be a result of the various operation waiting time thresholds in the different studies. Calculating operation waiting time as a continuous independent variable could be a solution to this discrepancy. Nonetheless, our study demonstrated no statistical difference in the 30-day mortality rate across the early and delayed groups, even though a prolonged length of hospital stay was found, as shown in the previous studies [21,22]. Previous studies reported an increased risk of infection in patients who underwent arthroplasty [26,27]. However, our study revealed no statistically significant difference in comorbidities between the internal fixation and arthroplasty groups.

A possible “weekend effect” was found in our analysis, despite the fact that previous large cohort investigations showed inconclusive evidence. The weekend effect is defined as a less favored outcome in patients who were admitted to hospital during the weekend. One study analyzed 38,020 geriatric patients who had acute hip fracture in Denmark and showed no outcome alteration in patients admitted during weekends, holidays, or working days [28]. In contrast, another analysis in Denmark that collected the data of 25,305 elderly patients with hip fracture found increased probability of surgical delay and 30-day mortality when patients were admitted during the weekend [29]. The weekend effect was also observed in our study. Although 74% of geriatric patients received surgery within 36 h, approximately 52.6% in the delayed surgical group were admitted to our hospital on weekends with statistical significance (*p* < 0.05). A study by Cha et al. [22] found that the causes of surgical delays were weekend or holiday admissions (27.2%) or delayed emergency department admission (24.6%). Providing sufficient resources for weekend and holiday operations could be a solution for this effect. Another possible solution is to identify high risk patients for foreseeable medical complications. Future studies should make an effort to identify vulnerable patients for an urgent operation.

There were some limitations in this study. First, this is a retrospective single-center study. Although patients’ baseline characteristics revealed no critical difference, unmeasured confounding factors and selection bias could affect the results. Moreover, our results may not be applicable under other hospital settings. Randomized control trials may provide stronger evidence for this issue, but may not be practical owing to ethical concerns. A large cohort study among Asian countries could be a reasonable solution. Second, although we excluded acute hip fracture patients who were transferred from other hospitals for operation, the operation waiting time may also be affected by how soon the patients entered the ER after the injury. We regard acute hip fracture as an incapacitating and painful situation for which patients would seek medical assistance immediately.

## 5. Conclusions

The results of the current study suggested that an operation waiting time threshold of 36 h was associated with increased risks of pneumonia, myocardial infarction, and heart failure in the included Asian geriatric patients, while an operation waiting time exceeding 36 h was not associated with 30-day mortality.

## Figures and Tables

**Table 1:** Baseline characteristics of patients who underwent hip fracture surgery divided into early and delayed surgery group by 36-h threshold.

		Early Surgery Group *n* = 827	Delay Surgery Group *n* = 291	All Patient *n* = 1118	*p*-Value
Operation wait time, mean ± SD		19.87 ± 6.9	55.79 ± 15.44	29.22 ± 19.55	
**Basic Characteristics**					0.143
Gender, *n* (%)	Male	246 (0.29)	100 (0.34)	346 (0.31)	
	Female	581 (0.71)	191 (0.66)	772 (0.69)	
Age, mean ± SD		80.69 ± 7.51	81.16 ± 7.66	80.81 ± 7.55	0.519
Hypertension, *n* (%)		301 (36)	110 (37)	411 (37)	0.67
Diabetes Mellitus, *n* (%)		319 (38)	132 (45)	451 (40)	<0.05 (0.042)
Hemoglobin, mean ± SD		11.76 ± 4.4	11.33 ± 2.1	11.65 ± 3.9	0.63
AST (aspartate aminotransferase), mean ± SD		28.56 ± 16.6	30.13 ± 20.5	28.96 ± 17.7	0.28
Estimated GFR ^a^, mean ± SD		61.51 ± 33.70	53.43 ± 27.57	59.41 ± 32.40	0.94
INR (international normalized ratio), mean ± SD		1.11 ± 0.96	1.12 ± 0.79	1.11 ± 0.92	0.76
**Emergency Room Characteristics**					
GCS, mean ± SD		14.96 ± 0.26	14.86 ± 0.89		<0.05
Level of Triage, *n* (%)					0.69
	Level 1	1 (0.1%)	1 (0.3%)	2 (0.2%)	
	Level 2	72 (8.7%)	30 (10%)	102 (9%)	
	Level 3	745 (90%)	256 (88%)	1001 (90%)	
	Level 4	9 (1%)	4 (1.4%)	13 (1.2%)	
ISS, mean ± SD		9.11 ± 1.39	9.21 ± 1.02	9.14 ± 1.31	0.43
Date of Emergency Room visit, *n* (%)					<0.05
	Weekdays	650 (78.6%)	138 (47.4%)	788 (70.5%)	
	Weekends	177 (21.4%)	153 (52.6%)	330 (29.5%)	
**Fracture Characteristics**					
Fracture Type, *n* (%)					0.428
	Femoral neck	418 (50.5)	160 (55)	578 (51.7)	
	Intertrochanteric	378 (45.7)	121 (41.6)	499 (44.6)	
	Subtrochanteric	31 (3.7)	10 (3.4)	41 (3.7)	
Operation Type, *n* (%)					0.157
	Internal Fixation	508 (61)	165 (57)	673 (60)	
	Arthroplasty	319 (39)	126 (43)	445 (40)	
Timing of Operation, *n* (%)					0.521
	Working hours	774 (93.6)	274 (94.2)		
	Evening	44 (5.3)	12 (4.1)		
	Overnight	9 (1.1)	5 (1.7)		

^a^ Estimated GFR: Estimated glomerular Filtration Rate was calculated by using Modification of Diet in Renal Disease (MDRD) equation. GCS: Glasgow coma scale. ISS: injury severity score.

**Table 2:** Clinical Outcomes of Early and Delayed Surgical Groups.

		Early Surgery Group *n* = 827	Delay Surgery Group *n* = 291	*p*-Value	Multivariate Analysis *
Pneumonia					
	30 days	36 (4.4%)	23 (7.9%)	<0.05	<0.05
	90 days	51 (6.2%)	31 (10.7%)	<0.05	<0.05
	365 days	106 (12.8%)	50 (17.2%)	0.07	0.13
Acute Myocardial Infarction					
	30 days	25 (3%)	21 (7.2%)	<0.05	<0.05
	90 days	47 (5.7%)	27 (9.3%)	<0.05	<0.05
	365 days	72 (8.7%)	35 (12%)	0.10	0.11
Heart Failure					
	30 days	126 (15.2%)	78 (26.8%)	<0.05	<0.05
	90 days	134 (16.2%)	83 (28.5%)	<0.05	<0.05
	365 days	162 (19.6%)	95 (32.6%)	<0.05	<0.05
Deep Vein Thrombosis					
	30 days	8 (1%)	6 (2.1%)	0.22	0.14
	90 days	9 (1.1%)	7 (2.4%)	0.15	0.09
	365 days	14 (1.7%)	10 (3.4%)	0.09	0.07
Pulmonary Embolism					
	30 days	3 (0.4%)	0 (0%)	0.57	0.31
	90 days	4 (0.5%)	0 (0%)	0.58	0.24
	365 days	5 (0.6%)	0 (0%)	0.18	0.19
Mortality (30 days)		3 (0.4%)	3 (1%)	0.19	
Length of Stay		7.4 (3.6)	9.6 (6.1)	<0.05	
Operation time		115.4 (37.1)	104.9 (40.0)	0.40	
Operation blood loss		211.3 (227.5)	177.8 (148.3)	0.26	

* Multivariate analysis adjusting for gender, age, ISS, triage level, and GCS.

**Figure A1:**
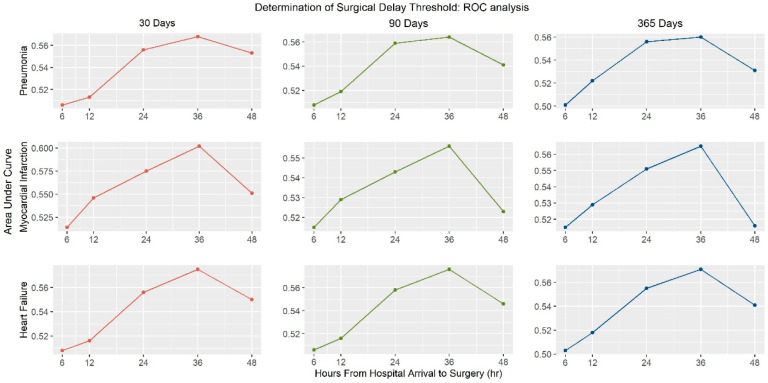
ROC (receiver operating characteristic) analysis in each comorbidity showed a significant difference across early and delayed groups. The analysis revealed that the AUC (area under curve) was the highest when the threshold was 36 h.

## Data Availability

The data are not publicly available due to restrictions regarding the Ethical Committee Institution.
